# Co-design, implementation, and evaluation of plain language genomic test reports

**DOI:** 10.1038/s41525-022-00332-x

**Published:** 2022-10-22

**Authors:** Gemma R. Brett, Aisha Ward, Sophie E. Bouffler, Elizabeth E. Palmer, Kirsten Boggs, Fiona Lynch, Amanda Springer, Amy Nisselle, Zornitza Stark

**Affiliations:** 1 Victorian Clinical Genetics Services, Murdoch Children’s Research Institute, Melbourne, VIC Australia; 2 The University of Melbourne, Melbourne, VIC Australia; 3 Australian Genomics, Melbourne, VIC Australia; 4 Sydney Children’s Hospitals Network, Sydney, NSW Australia; 5 The University of New South Wales, Sydney, NSW Australia; 6 Murdoch Children’s Research Institute, Melbourne, VIC Australia; 7 Monash Genetics, Monash Health, Melbourne, VIC Australia; 8 Monash University, Melbourne, VIC Australia

**Keywords:** Genetic testing, Genetic testing, Genetic counselling, Genetic testing, Patient education

## Abstract

Understanding and communicating genomic results can be challenging for families and health professionals without genetic specialty training. Unlike modifying existing laboratory reports, plain language genomic test reports provide an opportunity for patient/family-centered approaches. However, emerging examples generally lack co-design and/or evaluation in real-world settings. Through co-design involving patient groups, plain language experts, educators, and genetic health professionals, plain language genomic test report templates were produced for common test outcomes in rare diseases. Eight plain language genomic test report templates were developed. These reports were piloted and evaluated as part of a national pediatric ultra-rapid genomic testing program. Family and genetic health professional experiences with report layout, content, and use were explored using surveys. Of 154 families and 107 genetic health professionals issued with reports, 51 families and 57 clinicians responded (RR = 33% and 53%, respectively). Most families (82%) found their report helpful in understanding the result. Reports were shared by 63% of families, predominantly with family members (72%), or health professionals (68%). Clinicians (15%) adapted the reports for other settings. Through co-design, plain language genomic test reports implemented in a real-world setting can facilitate patient/family and caregiver understanding and communication of genomic test purpose, outcome, and potential clinical implications.

## Introduction

Understanding and communicating genomic test results can be challenging for families and health professionals without genetic specialty training. Patients and parents/guardians are increasingly provided laboratory genomic test reports by health professionals and/or via online secure medical records. However, the technical content and language of laboratory reports may cause confusion or uncertainty regarding clinical relevance and implications of genomic test outcomes, even amongst health professionals^1^. Accurate plain language resources to assist genomic test result interpretation may be difficult or impossible to locate, particularly for individuals with low literacy and/or for ultra-rare diseases. As health professionals with varying genetics knowledge and experience are increasingly ordering and/or interpreting genomic test outcomes^2–5^, plain language summaries may facilitate meaningful understanding and communication of results^6–13^. Understanding genomic test purpose, outcome, and potential clinical implications has the potential to enhance patient/family experiences, satisfaction, and engagement, which may lead to better health outcomes. Patients and their families have expressed a desire for plain language genomic test reports providing information consistent with clinical interpretations and recommendations available to health professionals, both for their own understanding and to communicate information with caregivers (e.g., physicians, allied health professionals, teachers, etc.)^7,9,14^.

The concept of plain language genomic test result summaries to facilitate and promote patient/family engagement and understanding is not new, however, there remains a lack of published evidence or guidelines regarding appropriate design approaches, layout, content, or use^6,10,11^. Professional guidelines regarding reporting genomic test outcomes primarily define laboratory report content, commonly revised as laboratories adapt to technological advances^15^. Rather than modifying existing laboratory reporting guidelines, it is necessary to consider appropriate methodologies for informing plain language genomic test report development and ensuring patient/family needs are met. Several studies recommend patients and families are directly involved in the design, to ascertain optimal report layout and content through co-design processes^6,10–13^. Although design approaches in commercial settings commonly involve co-design with target audiences, there remain few peer-reviewed studies detailing genomic test report co-design with non-specialist end-users^7,11,12,16^. Developing a completely revised patient/family-friendly report has been recommended, as a plain language resource provided alongside laboratory reports where appropriate^6^. It has also been recommended that co-design processes be utilized to develop plain language genomic test report templates containing key general information that can be personalized^11,13^. Considering the complexity of genomic testing and outcomes, plain language genomic test report development requires careful exploration and consideration of patient/family preferences for report layout, content, and use in order to maximize result comprehension and communication.

While emerging literature explores plain language genomic test report development and potential use in clinical settings^6,7,9–11,13,17^, published reports generally lack co-design and/or are limited to single genetic conditions. Additionally, published evaluations of plain language genomic test reports have primarily used hypothetical scenarios. Considering the heightened emotions often involved in genomic testing, it is essential patient/family resources are evaluated in real-world settings. This study aimed to co-design, pilot nationally in an acute pediatric setting, and evaluate the real-world use of plain language genomic test reports.

## Results

### Plain language genomic test report templates

**Fig. 1:**
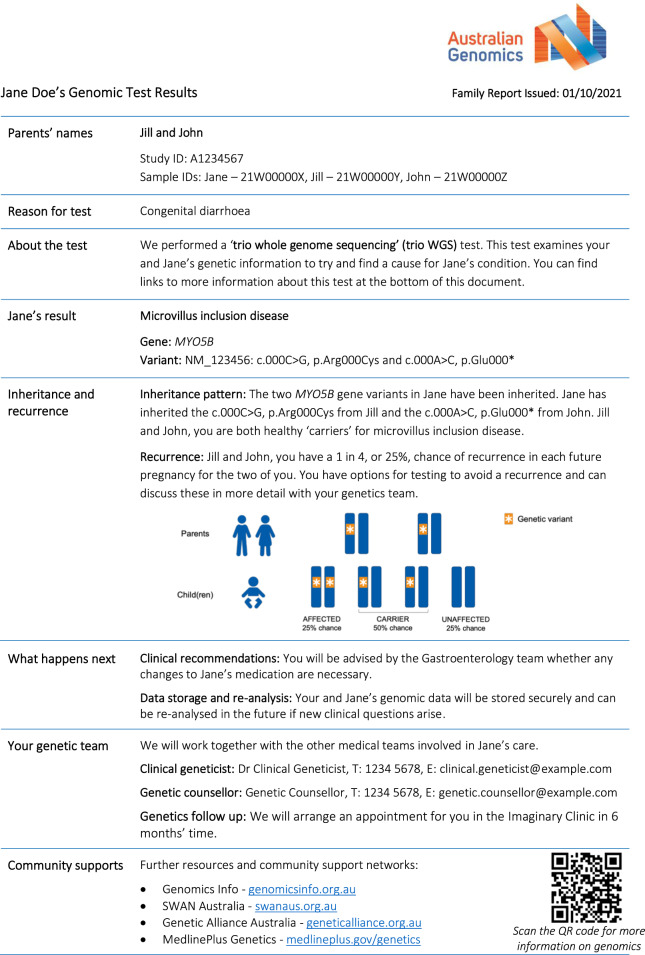
Example pre-filled “family report” (autosomal recessive template). This “family report” includes sections stating the genomic test type and result; inheritance and recurrence information with pictorial representation; follow up plans and information resources.

Co-design consultation feedback included suggestions regarding the amount of information, layout, and use of color to distinguish concepts and highlight key messages. Ultimately, eight plain language genomic test report templates were produced (report templates available in Supplementary Material). Six templates for where a diagnosis was achieved; de novo autosomal dominant, inherited autosomal dominant, autosomal recessive, X-linked inherited, X-linked de novo, and mitochondrial. Two templates for where a diagnosis was not achieved; variant(s) of unknown significance with high clinical relevance (i.e., strongly suspected to be causing the phenotype), and uninformative result (i.e., no variants reported). Test limitations were only included for uninformative results. As disease-specific resources are often absent in rare disease settings, sections regarding “what happens next” and “community supports” were pre-filled with general information. During the study period, 159 family reports were issued (pre-filled report example in Fig. 1): 31 de novo autosomal dominant, three inherited autosomal dominant, 23 autosomal recessive, three X-linked inherited, 17 variant of unknown significance, and 82 uninformative reports.

### Evaluation sample

Of 159 families issued a family report, three did not provide an email address, one was excluded on clinician’s recommendation, and one was not sent survey invitations due to an administrative error. One third of 154 families responded (*n* = 51; response rate 33%). Family respondents were the mother (*n* = 40, 78%) or father (*n* = 11, 22%) of the child undergoing urGS; the majority had post-secondary education (*n* = 34/50, 68%); greater than one third had income in the highest centiles (*n* = 19/49, 39%) (further family demographics in Supplementary Table 1). Most did not have prior experience with genetic conditions (*n* = 38/47, 81%). Although half had previous experience with genetic testing (*n* = 22/46, 48%), this was almost exclusively prenatal and/or reproductive testing (*n* = 21/22, 95%). Five (10%) did not recall or were unsure if they received the family report and were exited from the survey prior to questions regarding layout, content, and use. An additional four (8%) chose to exit the survey at this point, leaving 42 surveys included in the analysis.

Of 107 clinicians, 57 responded (response rate 53%). Clinician respondents were medical geneticists (*n* = 27, 47%), medical genetics trainees (*n* = 12, 21%), or genetic counselors (*n* = 17, 30%), with one additional respondent declining to specify their profession (further clinician demographics in Supplementary Table 2). Four (7%) reported they had not used a family report and were exited from the survey prior to questions regarding layout, content, and use, leaving 53 surveys included in the analysis.

**Fig. 2:**
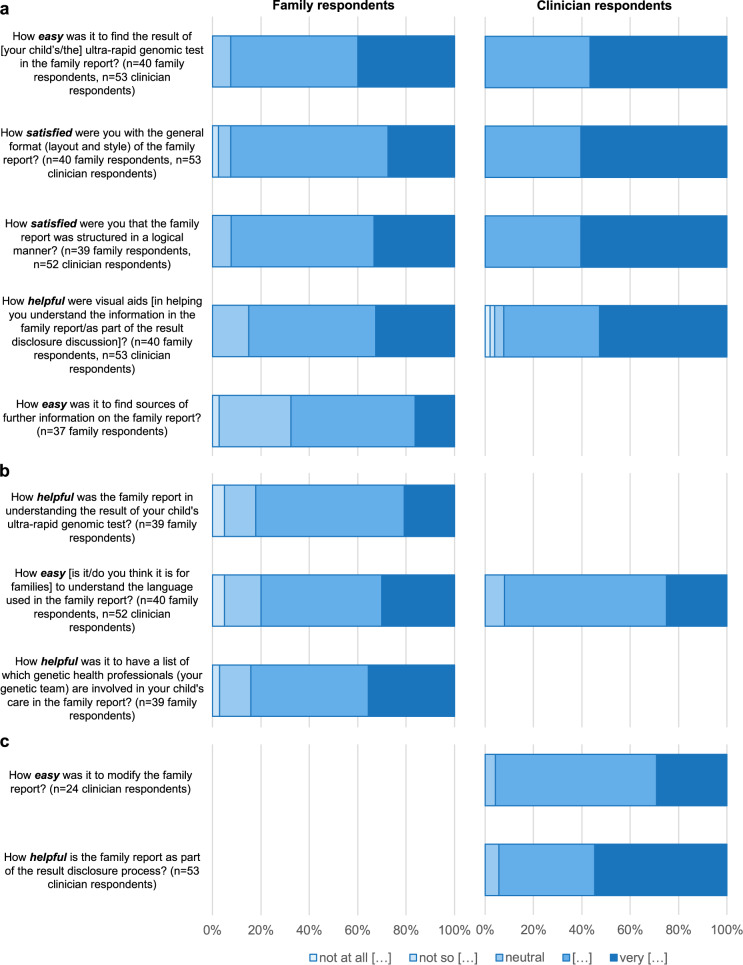
Family and clinician responses to five-point Likert scale questions. **a** Layout of “family reports”. **b** Content of “family reports”. **c** Use of “family reports”. The qualifying term represented by […] in legend is in ***bold/italics*** within the respective question.

Some respondents skipped questions; therefore sample size is included throughout. Figure 2 displays five-point Likert scale responses regarding layout, content, and use (means reported in Supplementary Table 3).

### Preferences for receiving and understanding information

Families reported they generally find it helpful if tables and graphs are used to explain parts of a story (*n* = 35/39, 90%). Percentages were overwhelmingly preferred by families (*n* = 38/39, 97%) compared with using only words when explaining predictions. While over half of families reported it easier to understand percentages compared with fractions (*n* = 21/39, 54%), many others reported no difference (*n* = 16/39, 41%).

### Layout of “family reports”

The result was easy to find in the report for the majority of families (*n* = 37/40, 93%) and all clinicians (*n* = 53/53, 100%). Similarly, layout and style satisfied most families (*n* = 37/40, 93%) and all clinicians (*n* = 53/53, 100%). Visual aids (e.g., pictures, bolded text, section headings, etc.) were helpful for the majority of families (*n* = 34/40, 85%), with one respondent commenting “*bolded sections/headings helpful for key information*” and another suggesting “*different colors for different sections of the report*”. Most clinicians (*n* = 49/53, 92%) found visual aids helpful as a part of result disclosure discussions, with some commenting positively regarding overall visual layout, e.g., “*excellent formatting and graphic design, which is key to enabling easy access to the information*”, and others specifically regarding inheritance pattern graphics, e.g., “*visual aids describing inheritance are particularly helpful*“. Two-thirds of families reported it easy to find sources of further information within the report (*n* = 25/37, 68%). Most families (*n* = 36/39, 92%) and all clinicians (*n* = 52/52, 100%) were satisfied the report was structured in a logical manner.

### Content of “family reports”

Most families found the report helpful (*n* = 32/39, 82%) in understanding their child’s test result, and accurately recalled whether their child received a diagnosis, incomplete diagnosis, or no diagnosis (*n* = 35/41, 85%). From the one third of families (*n* = 15/41, 36%) whose child received a diagnosis or incomplete diagnosis, and therefore recurrence risk was on their report, most (*n* = 11/15, 73%) accurately recalled their recurrence risk. Of families whose report included limitations of testing (uninformative result template), two-thirds recalled their report explained test limitations (*n* = 14/21, 67%).

Families reported the language used was easy to understand (*n* = 32/40, 80%) and medical terms were explained in a clear manner (*n* = 37/40, 93%). Similarly, clinicians thought it would be easy for families to understand the language used (*n* = 48/52, 92%), and medical terms were explained in a sufficiently clear manner (*n* = 49/53, 92%), with several comments such as the report was “*clear, easy to read and concise*”.

Most families found it helpful that reports listed genetic health professionals involved in their child’s care (*n* = 33/39, 85%). When asked if there were other types of information they would find helpful, two families whose children did not receive a diagnosis expressed a desire for details of all conditions/genes reviewed in the analysis, e.g., “*I understand that can be difficult to provide this information, but I would like to understand which genetic conditions my child was assessed against… I don’t know which conditions have been excluded*“. Families (*n* = 38/40, 95%) and clinicians (*n* = 47/53, 89%) generally felt the reports did not contain unnecessary information. However, some clinicians suggested laboratory identification numbers could be omitted or replaced with date of birth, and/or emphasized community resources should be reviewed and updated by clinicians where appropriate.

### Use of “family reports”

Most clinicians (*n* = 50/53, 94%) found the reports helpful as part of result disclosure, using it at the start (*n* = 5/53, 9%), during (*n* = 26/53, 49%) and/or end (*n* = 44/53, 83%) of the consultation. Comments from clinicians (*n* = 20/53, 38%) highlighted the clinical utility of the report, particularly regarding facilitating families’ understanding during result disclosure, and usefulness as a written/visual record for families to take-home (Table 1). Some clinicians (*n* = 25/53, 47%) reported modifying family report content, such as adding management plans, specific support groups, and/or online resources, and most felt this was an easy modification process (*n* = 23/25, 92%).

**Table 1:** Illustrative quotes from clinician respondents regarding the use of “family reports”.

***Use during result disclosure***
*Much better than scribbling things on paper in front of the family, or trying to guide them through the complex language of laboratory reports*.
*Genetics is so scientific and abstract, having a physical item in their language, clearly spelt out and* “*bespoke’*” *is so powerful. Thanks for this!!*
*For some families it works best as a take-home record at the end of a results consultation, however others seem to follow the discussion better if they can glance down at the report*.
*Very helpful to be able to leave the family with a clear, concise report after what is often a very complex appointment in a high stress situation for the family*
*For informative results, the family report can be useful during the results disclosure session as a visual guide for families to follow the discussion. For uninformative results, the family report is more useful as a take-home record at the end of the consultation*.
***Distribution***
*…very useful in helping other specialists who were not at the result disclosure meeting to be able to understand the results*
*[I would distribute to] all involved clinicians as I think everyone struggles to understand the main report. Assists in communication and care coordination*
*… helpful for a brief summary of results for non-genetics health professionals, easy to access in patient record*
***Use beyond the acute pediatric setting***
*I believe it should be the basis of all future genetics consultations … as a supplement to genetics letters and information giving*
*I would also use the format to guide a simple language family report in the future outside of acute care. I had thought about that but not done it yet!*
*I have taken inspiration from these reports to layout reports for other research projects. They have not been used as a direct template, but the flavor of the report has been helpful to generate other reports*
*I think these should be adopted much more broadly as they really help families to understand and retain the information in a way that is practical to them*.
*Would be helpful for [molecular genetics] labs to incorporate family reports, so that a family report is routinely generated as part of genomic testing (similarly, so that clinicians can edit as appropriate)*

The majority of families felt confident using the report to explain their child’s urGS result to someone else, such as family and friends (*n* = 32/39, 82%), and explaining to other family members whether they have a chance of being affected themselves and/or having a child with the same condition (*n* = 32/38, 84%). Most families who did not feel confident explaining the recurrence risk did not receive a diagnosis for their child and therefore did not have a reported recurrence risk (*n* = 5/6, 83%). Approximately half of the families planning or potentially planning more children felt the report had enough information for family planning (*n* = 12/23, 52%). Most families who felt the report did not have enough information for family planning did not receive a diagnosis for their child and therefore did not have a reported recurrence risk (*n* = 10/11, 91%), with the one remaining respondent who did have a recurrence risk expressing a desire for the report to “*outline all options for future family planning*”.

Many families (*n* = 25/40, 63%) shared their report with health professionals (*n* = 17/25, 68%) (e.g., primary care physicians, medical specialists, nurses, and allied health professionals), and/or family members (*n* = 18/25, 72%) (e.g., siblings, parents, aunts, uncles, and cousins), and/or friends (*n* = 9/25, 36%). Two respondents, both of whom received a diagnosis for their child, stated they shared their report with over 50 friends in addition to their extended families, with one commenting, “*We appreciated the extended medical report as well, and having that option was good for us. We shared the family report extensively with our friends and family who we have been updating on our child’s condition. They are from diverse backgrounds and those who engaged with the report were generally understanding of its content*”. Clinicians (*n* = 16/53, 30%) also reported distributing the reports, predominantly to specialist medical teams, social workers, and/or primary care physicians involved in the child’s care, with several commenting on the potential utility for health professionals without genetic specialty training, and usefulness of adding the family report to medical records (Table 1).

Eight clinicians (15%) reported adapting and using the report templates in other settings, including the return of genomic testing results for outpatients, inpatients, and/or research participants. Some clinicians also stated they are requesting their local laboratories and/or clinical genetics services to adopt similar family reports more broadly (Table 1).

## Discussion

As demonstrated here, plain language genomic test reports can be successfully co-designed and nationally implemented in a real-world setting to facilitate family and clinician understanding and communication of genomic test results across a broad range of rare disease test outcomes. Despite the added challenges of result disclosure in acute pediatrics, there were high levels of family and clinician satisfaction with layout, content, and language, with family reports being shared broadly and the templates adapted for use in other settings.

Co-designing plain language report templates with end-users emphasized patient/family experience is of equivalent value to information content^18^. Distinct lived experiences, health literacy, and preferences for receiving and interpreting information can make it challenging to develop reports which effectively communicate information to diverse recipients^6^. Co-design processes enable the identification and resolution of issues with report utility, hence overcoming mismatches between information deemed essential by genetics professionals and patients/families^10,11^. In this study, the number of templates developed reflects end-users’ desire for result representation tailored to genomic test outcomes, highlighting the need for careful co-design when ascertaining optimal representation of complex and varied reporting scenarios. Templates developed here to expand upon the small number of published plain language genomic test report examples, adding a set with demonstrated use in a real-world setting. Although using several templates may have practical considerations for implementation in clinical laboratory settings, meeting patient/family needs should be paramount when approaching system change.

The layout of the plain language genomic test report templates aligned with principles historically applied to laboratory reports intended for clinician use: synoptic reporting frameworks and patient narratives^15^. User feedback in this study supports emerging evidence that partitioning content under clear section headings, per synoptic reporting frameworks, satisfies families and clinicians alike^7,10^. It is well established that results should feature prominently in reports^6,10^. Both families and clinicians in this study indicated test results were easy to find, with the mean Likert scores for respondents here similar to means reported in a study comparing user-centered reports with standard laboratory reports^11^. Family and clinician satisfaction with the report structure indicates applying the patient narrative format, whereby content reflects the order of events experienced by patients, was appropriate. The strong family preference for tables and graphs was consistent with suggestions in the literature that visual representations can improve comprehension^6^. Visual aids were helpful for families and clinicians, as previously reported^7,19^. However, there remains minimal peer-reviewed evidence regarding patient/family preferences for the design and use of visual aids such as graphs and/or diagrams in plain language reports, and further research is needed to determine the optimal visual presentation of complex genetic concepts to lay audiences. Family reports in this study were ideally kept to a single page, however, patients/families may not consider this a priority provided appropriate section headings are used^7^, and the document remains simple and easy to navigate^10^.

Key components of plain language test result summaries include the meaning of the result, the next steps to be taken, and where to find further information and/or support^7,10^. Templates developed here followed recommended measures of presenting information in a personal tone, using first-person language and child/parent names, and avoiding jargon (e.g., omitting variant classifications)^1,7,8,11,16^. Responses indicating mixed family preferences for percentages versus fractions may support the established practice of displaying percentages and fractions together^7^. Survey responses indicate content and language were generally successful in helping families understand genomic test results, with mean Likert scores similar to means reported in a large-scale evaluation using a hypothetical scenario^11^. However, it is important to acknowledge comments from families presented with information about genomic testing limitations in the absence of a diagnosis. In future, dynamic online resources may facilitate access to personalized plain language information regarding genes analyzed and associated conditions^20^. Family and health professional desires for information regarding “next steps”^1,7,8,11,16^ may be more easily satisfied in clinical settings where templates can be pre-filled with disease-specific clinical guidance and community supports regarding commonly encountered diagnoses (e.g., neuromuscular clinics). This may be harder to achieve in settings where more heterogenous groups of patients are tested, and where an ultra-rare diagnosis may replace the diagnostic odyssey with a new odyssey^21,22^.

Plain language genomic test reports are anticipated to have broad utility as a result disclosure communication tool, a patient/family reference (given volume of information and heightened emotions during result disclosure), and a resource to be shared with family, friends, healthcare providers, and in non-healthcare settings^7–10,17^. Clinician responses highlighted the clinical utility of the family report, which was most often distributed after result disclosure for reference. Families and clinicians shared the family report widely. Sharing these reports may empower families to communicate with health professionals regarding genomic test purpose and outcome, may serve to disseminate information relevant to family members' health, and may relieve the communication burden of keeping friends or family “up to date”^7^. Additionally, plain language reports have potential utility for facilitating family communication regarding cascade testing, where appropriate. Two families here notably reported sharing their report with over 50 individuals for this purpose. Parents in hypothetical situations reportedly anticipate sharing plain language genomic test reports in other non-healthcare settings (e.g., with educators, therapists, support workers, etc.), to empower and encourage understanding of their child’s rare condition^7,9^. While such findings were not reflected in real-world family experiences reported here, this may be due to the Acute Care Genomics study cohort being primarily critically ill infants not yet engaged with comparable non-healthcare settings^23^.

This real-world evaluation was limited to the experiences of families and genetic health professionals who received/used personalized “family reports” summarizing urGS results in acute pediatrics. Most families had no previous experience with genetic conditions or genetic testing beyond prenatal and/or reproductive testing. There is emerging evidence that health professionals without genetic specialty training value receiving plain language reports alongside standard genomic test reports^8^. However, the identities of secondary users of the plain language reports, such as health professionals, support workers, family members, and friends, were not known to the study investigators, therefore the scope of this study limited respondents to parent/guardians and their genetic health professionals. Despite good response rates, small sample sizes limited statistical power. Therefore, it was not possible to examine potential differences based on demographics, such as whether satisfaction was related to factors other than the family report itself (e.g., receiving a diagnosis)^9^. It is not known whether all families received their personalized reports as part of the resulting disclosure. The Acute Care Genomics study did not exclude children based on socioeconomic status or parent/guardian English language skills. However, as the majority of family respondents had post-secondary education and reasonable financial means, their experiences may not be representative of the general population. Also, individuals invited to co-design the plain language templates were prioritized to have lived experiences of genetic conditions and receive genetic/genomic test results, rather than to be representative of all sectors of the community. Health literacy and numeracy were not evaluated in this study. The reading level of plain language in the report templates was determined via plain language specialist review, however, parents/guardians with low English language skills or reading literacy may have difficulty understanding written information in plain language genomic test reports. Experiences of individuals unable to complete the survey in written English language were not captured, therefore reducing the diversity of experiences reported. This study provides a foundation for future research into plain language genomic test report use in varied healthcare settings, with diverse end-users.

Practical challenges remain for wider implementation of plain language genomic test reports in laboratories and clinical genetics services. In this study, the report templates were pre-filled manually, however, deployment at scale in clinical laboratories would likely require automated processes similar to the generation of traditional laboratory reports. While it has been hypothesized plain language genomic test reports are of value to patients/families regardless of test outcome^7^, data reported here indicate specific informational needs may exist for patients/families who did not receive a diagnosis; therefore, it is recommended future studies explore the needs of this group. As society increasingly moves toward electronic resources, electronic deployment may better serve patients/families^7^, potentially via quick response (QR) codes and/or hosted within patient-facing secure medical record access^17^. This may facilitate interactive and/or dynamic personalized information, and theoretically be more agile for translating into other languages. Further studies are recommended to refine plain language genomic test report use in varied healthcare and non-healthcare settings, and to explore the long-term use and utility of such reports as a resource for patients/families and whomever they choose to share their report with.

Inherent to developing plain language genomic test report content is the challenge of summarizing and presenting complex information in a volume and language accessible to patients/families and health professionals without genetic specialty training^6,9–11^. It remains important for clinicians to be mindful that individual experiences and personal theories of risk may affect the understanding of test results^6,24^. Hence, plain language genomic test reports are designed to enhance patient/family understanding of genomic test outcomes and implications, and are not intended to replace laboratory reports or result disclosure discussions with appropriately trained health professionals^6,9–11^. The findings here indicate that, through co-design, plain language genomic test reports implemented in a real-world acute pediatric setting may facilitate patient/family and caregiver understanding and communication of genomic test purpose, outcome, and potential clinical implications. Already, the report templates developed in this study are being adapted for broader clinical and research use, highlighting the need for wider implementation of understandable genomic result summaries in diverse areas of genomics.

## Methods

### Context

The Acute Care Genomics study was a national ultra-rapid genomic diagnosis program for critically ill infants and children admitted to hospitals with suspected genetic conditions^23^. Patients were recruited prospectively from 17 hospitals, including all children’s hospitals in Australia. Ultra-rapid genome sequencing (urGS) was performed, as trio where possible, with results issued in <5 calendar days and generally disclosed to parents/guardians prior to hospital discharge. Pre- and post-test counseling was provided by genetic health professionals. Participants provided voluntary, informed written consent via ethically approved paper or electronic forms. Ethics approval was granted by the Royal Melbourne Hospital Human Research Ethics Committee (HREC/16/MH/251) and The Royal Children’s Hospital Melbourne Human Research Ethics Committee (HREC/78910/RCHM-2021).

### Co-design of plain language genomic test report templates

A scoping review identified limited publications regarding plain language summaries of genomic test results and/or co-design of plain language genomic test reports^10,11^. Additional plain language summaries used in clinical genetics practice were obtained via personal correspondence with international experts. Common elements included synoptic reporting frameworks (i.e., partitioning content under section headings), and patient narrative formats (i.e., content reflecting the order of events that patients experience). Using these elements, the authors drafted separate one-page plain language genomic test report templates for de novo autosomal dominant, autosomal recessive, and uninformative results. Feedback on the draft templates was sought from Acute Care Genomics study investigators (*n* = 27) via email. Consumer (*n* = 19) feedback was sought via email and presentation at meetings with the Australian Genomics Community Advisory Group, Queensland Genomics Health Alliance Community Advisory Group, and Syndromes Without A Name Australia. Draft templates were modified and improved through an iterative co-design process where feedback was provided by the study investigators and consumer groups regarding layout, content, language, and utility. Plain language specialist review evaluated the reading level of text and ensured reports were succinct and easy to read. Final templates were developed for common genomic test outcomes in rare diseases.

### Deployment of “family reports” in acute pediatrics

Plain language genomic test reports were issued for Acute Care Genomics study participants receiving urGS results (Melbourne Health HREC/16/MH251). Alongside laboratory report issues, the study team pre-filled relevant templates with genomic test results. Pre-filled reports were electronically distributed to the primary involved medical geneticist, medical genetics trainee (where relevant), and/or genetic counselor. Recipients were encouraged to replace pre-filled general information with personalized information where possible, including genetics team contact details, follow-up arrangements, and/or community supports, ideally keeping reports to one page. It was recommended that personalized plain language genomic test reports, called “family reports”, be provided to parents/guardians as part of result disclosure, likely as a printed copy during discussion and/or in portable document format (PDF) emailed afterwards.

### Evaluation of “family report” use in acute pediatrics

Two complementary online surveys were designed with questions tailored to family perspectives (Melbourne Health HREC/16/MH251) or clinician perspectives (Royal Children’s Hospital HREC/78910/RCHM-2021) regarding family report layout, content, and use (survey tools available in Supplementary Material). The inclusion criteria for “family respondents” were families issued a family report through the Acute Care Genomics study from October 2020 to November 2021. Exclusion criteria were families who withdrew from the study, requested not to receive surveys, did not provide an email address, or, if involved, genetic health professionals indicated the family was highly distressed. The inclusion criteria for “clinician respondents” were genetic health professionals caring for patients undergoing urGS via the Acute Care Genomics study.

Survey domains and questions were informed by survey tools from Recchia et al.^11^, Brett et al.^14^, and Nisselle et al.^25^. Survey questions were mapped to domains of personal characteristics, layout, content, and use (see Supplementary Table 4). Both surveys included a family report example image; respondents who did not recall receiving (families) or using (clinicians) a family report were exited from the survey. Custom “family survey” questions explored demographics, prior experience with genetic conditions and testing, and preferences for receiving and understanding information. Custom “clinician survey” questions explored Acute Care Genomics study experience and family report use. Both surveys included optional free-text response fields throughout.

Family survey invitations were sent 12 weeks after result disclosure and distributed to primary contact parent/guardian email addresses. The survey could only be completed once per family. If the survey was not completed, email reminders were sent after 2 and 4 weeks. Clinician survey invitations were sent via email, with three reminders over two months. Survey responses were collected and managed using REDCap electronic data capture tools hosted at the Murdoch Children’s Research Institute, Melbourne, Australia^26^. This paper reports data from responses to family invitations sent from March 2021 to February 2022, and clinician invitations sent from December 2021 to February 2022. Data were interrogated via descriptive statistical analyses conducted using Stata 17^27^. Likert scales are reported as proportions and means with standard deviations. Free-text responses were corrected for spelling and grammar, then reviewed for content and are reported as majority categories. They are also used illustratively to provide additional insight into respondents’ quantitative results, experiences, and opinions.

### Reporting summary

Further information on research design is available in the Nature Research Reporting Summary linked to this article.

## Supplementary information

Supplementary Material

Reporting Summary

## Data Availability

Plain language genomic test report templates and survey instruments are available as supplementary information. Detailed data regarding the survey responses are available upon individual request to gemma.brett@vcgs.org.au.
