# Supplementary material for: Co-design, implementation, and evaluation of plain language genomic test reports
Source: NPJ Genom Med. 2022 Oct 22;7:61. doi: 10.1038/s41525-022-00332-x (PMC9588009; doi:10.1038/s41525-022-00332-x)
Supplement: Supplementary file 1 — Supplementary Material [file 41525_2022_332_MOESM1_ESM.pdf]

Australian Genomics

Supplementary Notes 1. Plain language genomic test report templates

Brett GR, Ward A, Bouffler SE, Palmer EE, Boggs K, Lynch F, Springer A, Nisselle A, Stark Z.
Co-design, implementation, and evaluation of plain language genomic test reports.
npj Genomic Medicine 2022

Plain language genomic test report templates developed in the above study are enclosed in the following order:

- page 2: *de novo* dominant
- page 3: inherited autosomal dominant
- page 4: autosomal recessive
- page 5: X-linked inherited
- page 6: X-linked *de novo*
- page 7: mitochondrial
- page 8: variant(s) of unknown significance with high clinical relevance (i.e., strongly suspected to be causing the phenotype)
- page 9: uninformative result (i.e., no variants reported)

These plain language genomic test report templates were designed in Microsoft Word using Microsoft Forms fields. Use of these fields enables sections to be locked against changes to the template, while still enabling personalisation of relevant content.

When the document was 'unlocked', the templates were fully modifiable, allowing form fields to be added, edited or deleted.

When the document was 'protected' (enabled in the 'Developer' tab in Microsoft Word), the template content was 'locked' so only the fields in grey could be modified, along with the final sections of '*What happens next*', '*Your genetic team*', and '*Community supports*'.

Patient name's Genomic Test Results

Family Report Issued: dd/mm/yyyy

Parents' names

Parents' names

Study ID: A Testing Laboratory:
Sample IDs: Patient – 22W , Mum – 22W , Dad – 22W

Reason for test

Unexplained seizures in Patient

About the test

We performed a '**trio whole genome sequencing' (trio WGS)** test. This test examines your and Patient's genetic information to try and find a cause for Patient's condition. You can find links to more information about this test at the bottom of this document.

Patient's result

KCNQ2-related epileptic encephalopathy

Gene: *KCNQ2*
Variant: NM_000000: c.000C>G, p.Arg000Cys

Inheritance and recurrence

Inheritance pattern: Patient has not inherited the *KCNQ2* gene variant, it has occurred in him/her for the first time (it is *de novo*).

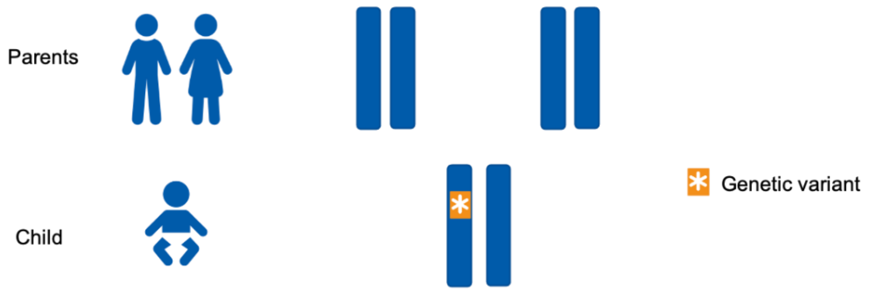

Recurrence: Parents' names, you have a low chance of recurrence in future pregnancies for this condition. You have options for testing to avoid a recurrence and can discuss these in more detail with your genetics team.

What happens next

Clinical recommendations: You will be advised by the Neurology team whether any changes to Patient's seizure medication are necessary.

Data storage and re-analysis: Your and Patient's genomic data will be stored securely and can be re-analysed in the future if new clinical questions arise.

Your genetic team

We will work together with the other medical teams involved in Patient's care.

Clinical geneticist:

Genetic counsellor:

Genetics follow up:

Community supports

Further resources and community support networks:

- Genomics Info - genomicsinfo.org.au
- SWAN Australia - swanaus.org.au
- Genetic Alliance Australia - geneticalliance.org.au
- MedlinePlus Genetics - medlineplus.gov/genetics

Scan the QR code for more information on genomics

Patient Name's Genomic Test Results

Family Report Issued: dd/mm/yyyy

Parents' names

Parents' names

Study ID: A Testing Laboratory:

Sample IDs: Patient – 22W , Mum – 22W , Dad – 22W

Reason for test

Unexplained cardiac arrest

About the test

We performed a '**trio whole genome sequencing' (trio WGS)** test. This test examines your and Patient's genetic information to try and find a cause for Patient's condition. You can find links to more information about this test at the bottom of this document.

Patient's result

Long QT syndrome

Gene: *SCN5A*
Variant: NM_000000: c.000C>G, p.Arg000Cys

Inheritance and recurrence

Inheritance pattern: The *SCN5A* gene variant in Patient has been inherited from mum/dad.

Recurrence: Parents' names, you have a 1 in 2, or 50%, chance of recurrence in each future pregnancy for the two of you. You have options for testing to avoid a recurrence and can discuss these in more detail with your genetics team. mum/dad, other members of your family are also at risk of Long QT syndrome.

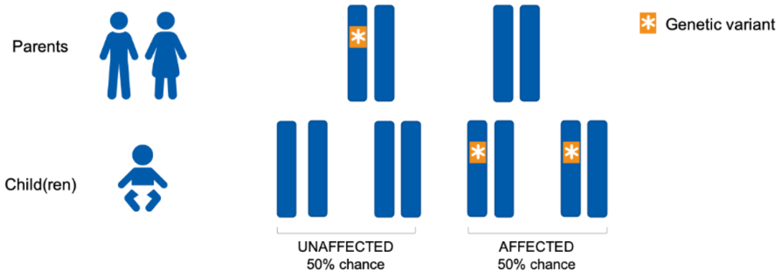

What happens next

Clinical recommendations: The Cardiology team will discuss management options with you. mum/dad, we will refer you to a cardiology specialist and we will discuss recommendations for testing other family members further.

Data storage and re-analysis: Your and Patient's genomic data will be stored securely and can be re-analysed in the future if new clinical questions arise.

Your genetic team

We will work together with the other medical teams involved in Patient's care.

Clinical geneticist:

Genetic counsellor:

Genetics follow up:

Community supports

Further resources and community support networks:

- Genomics Info - genomicsinfo.org.au
- SWAN Australia - swanaus.org.au
- Genetic Alliance Australia - geneticalliance.org.au
- MedlinePlus Genetics - medlineplus.gov/genetics

Scan the QR code for more information on genomics

Patient Name's Genomic Test Results

Family Report Issued: dd/mm/yyyy

Parents' names

Parents' names

Study ID: A

Testing Laboratory:

Sample IDs: Patient – 22W , Mum – 22W , Dad – 22W

Reason for test

Congenital diarrhoea

About the test

We performed a '**trio whole genome sequencing' (trio WGS)** test. This test examines your and Patient's genetic information to try and find a cause for Patient's condition. You can find links to more information about this test at the bottom of this document.

Patient's result

Microvillus inclusion disease

Gene: *MYO5B*

Variant: NM_000000: c.000C>G, p.Arg000Cys and c.000A>C, p.Glu000*

Inheritance and recurrence

Inheritance pattern: The two *MYO5B* gene variants in Patient have been inherited. Patient has inherited the c.000C>G, p.Arg000Cys from mum and the c.000A>C, p.Glu000* from dad. Parents' names, you are both healthy 'carriers' for microvillus inclusion disease.

Recurrence: Parents' names, you have a 1 in 4, or 25%, chance of recurrence in each future pregnancy for the two of you. You have options for testing to avoid a recurrence and can discuss these in more detail with your genetics team.

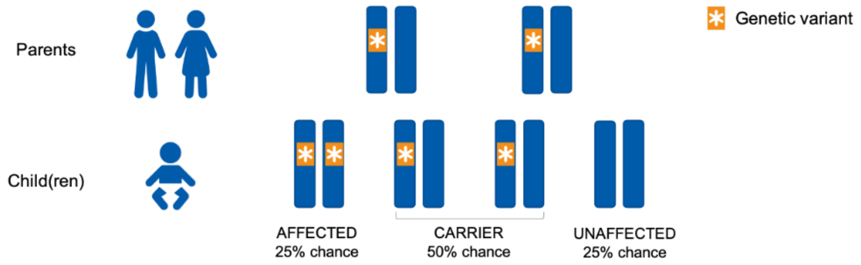

What happens next

Clinical recommendations: You will be advised by the Gastroenterology team whether any changes to Patient's medication are necessary.

Data storage and re-analysis: Your and Patient's genomic data will be stored securely and can be re-analysed in the future if new clinical questions arise.

Your genetic team

We will work together with the other medical teams involved in Patient's care.

Clinical geneticist:

Genetic counsellor:

Genetics follow up:

Community supports

Further resources and community support networks:

- Genomics Info - genomicsinfo.org.au
- SWAN Australia - swanaus.org.au
- Genetic Alliance Australia - geneticalliance.org.au
- MedlinePlus Genetics - medlineplus.gov/genetics

Scan the QR code for more information on genomics

Australian Genomics

Patient Name's Genomic Test Results

Family Report Issued: dd/mm/yyyy

Parents' names	Parents' names<br>Study ID: A Testing Laboratory:<br>Sample IDs: Patient – 22W , Mum – 22W , Dad – 22W
Reason for test	Seizures and developmental delay
About the test	We performed a '**trio whole genome sequencing' (trio WGS)** test. This test examines your and Patient's genetic information to try and find a cause for Patient's condition. You can find links to more information about this test at the bottom of this document.
Patient's result	Menke's disease<br>**Gene:** *ATP7A*<br>**Variant:** NM_000000: c.000A>C, p.Glu000*
Inheritance and recurrence	**Inheritance pattern:** Mum, Patient has inherited the *ATP7A* gene variant from you. Mum, you are a healthy 'carrier' for Menke's disease, and other members of your family may also be carriers.<br>**Recurrence:** Parents' names, you have a 1 in 4, or 25%, chance of recurrence in each future pregnancy for the two of you. You have options for testing to avoid a recurrence and can discuss these in more detail with your genetics team.<br>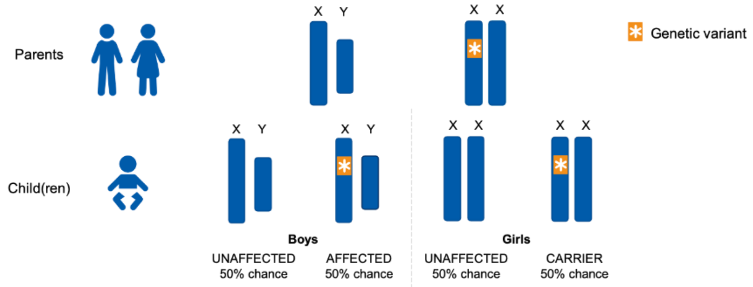
What happens next	**Clinical recommendations:** The Metabolic team will discuss management options with you.<br>**Data storage and re-analysis:** Your and Patient's genomic data will be stored securely and can be re-analysed in the future if new clinical questions arise.
Your genetic team	We will work together with the other medical teams involved in Patient's care.<br>**Clinical geneticist:**<br>**Genetic counsellor:**<br>**Genetics follow up:**
Community supports	Further resources and community support networks:<br>• Genomics Info - genomicsinfo.org.au<br>• SWAN Australia - swanaus.org.au<br>• Genetic Alliance Australia - geneticalliance.org.au<br>• MedlinePlus Genetics - medlineplus.gov/genetics<br>*Scan the QR code for more information on genomics*

Patient Name's Genomic Test Results

Family Report Issued: dd/mm/yyyy

Parents' names

Parents' names

Study ID: A Testing Laboratory:
Sample IDs: Patient – 22W , Mum – 22W , Dad – 22W

Reason for test

Seizures and developmental delay

About the test

We performed a '**trio whole genome sequencing' (trio WGS)** test. This test examines your and Patient's genetic information to try and find a cause for Patient's condition. You can find links to more information about this test at the bottom of this document.

Patient's result

Menke's disease

Gene: *ATP7A*
Variant: NM_000000: c.000A>C, p.Glu000*

Inheritance and recurrence

Inheritance pattern: Patient has not inherited the *ATP7A* gene variant, it has occurred in him/her for the first time (it is *de novo*).

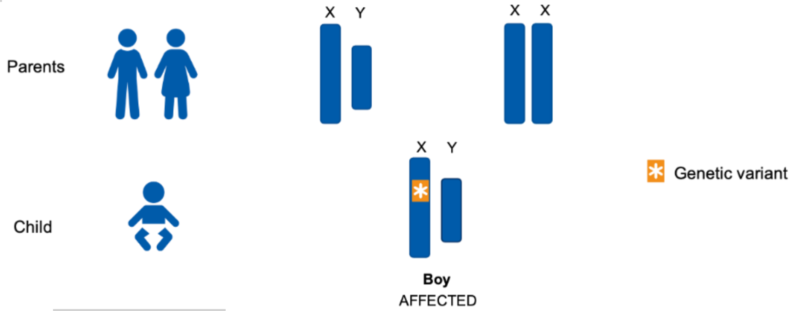

Recurrence: Parents' names, you have a low chance of recurrence in future pregnancies for this condition. You have options for testing to avoid a recurrence and can discuss these in more detail with your genetics team.

What happens next

Clinical recommendations: The Metabolic team will discuss management options with you.

Data storage and re-analysis: Your and Patient's genomic data will be stored securely and can be re-analysed in the future if new clinical questions arise.

Your genetic team

We will work together with the other medical teams involved in Patient's care.

Clinical geneticist:

Genetic counsellor:

Genetics follow up:

Community supports

Further resources and community support networks:

- Genomics Info - genomicsinfo.org.au
- SWAN Australia - swanaus.org.au
- Genetic Alliance Australia - geneticalliance.org.au
- MedlinePlus Genetics - medlineplus.gov/genetics

Scan the QR code for more information on genomics

Parents' names	Parents' names<br><br>Study ID: A Testing Laboratory:<br>Sample IDs: Patient – 22W , Mum – 22W , Dad – 22W
Reason for test	Suspected optic neuropathy
About the test	We performed a '**trio whole genome sequencing' (trio WGS)** test. This test examines your and Patient's genetic information to try and find a cause for Patient's condition. You can find links to more information about this test at the bottom of this document.
Patient's result	**Leber hereditary optic neuropathy**<br><br>**Gene:** *MT-ND4*<br>**Variant:** NC_000000: m.0000G>A, p.(Arg000His)
Inheritance and recurrence	**Inheritance pattern:**<br>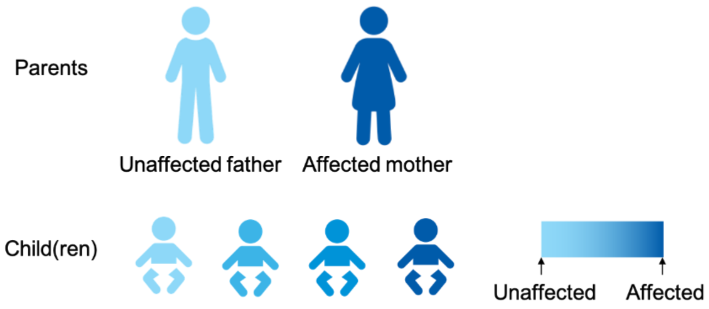<br>**Recurrence:**
What happens next	**Clinical recommendations:** The Metabolic team will discuss management options with you.<br><br>**Data storage and re-analysis:** Your and Patient's genomic data will be stored securely and can be re-analysed in the future if new clinical questions arise.
Your genetic team	We will work together with the other medical teams involved in Patient's care.<br><br>**Clinical geneticist:**<br><br>**Genetic counsellor:**<br><br>**Genetics follow up:**
Community supports	Further resources and community support networks:<br>• Genomics Info - genomicsinfo.org.au<br>• SWAN Australia - swanaus.org.au<br>• Genetic Alliance Australia - geneticalliance.org.au<br>MedlinePlus Genetics - medlineplus.gov/genetics<br><br>*Scan the QR code for more information on genomics*

Australian Genomics

Patient Name's Genomic Test Results

Family Report Issued: dd/mm/yyyy

Parents' names	Parents' names<br>Study ID: A Testing Laboratory:<br>Sample IDs: Patient – 22W , Mum – 22W , Dad – 22W
Reason for test	Hydrops
About the test	We performed a '**trio whole genome sequencing' (trio WGS)** test. This test examines your and Patient's genetic information to try and find a cause for Patient's condition. You can find links to more information about this test at the bottom of this document.
Patient's result	**No genetic diagnosis was made**<br>However, two gene variants were identified that may be indicative of Nemaline myopathy 2. At the present time, we do not have enough evidence to be certain they are responsible for Patient's condition.<br>**Gene:** *NEB*<br>**Variant:** NM_0000: c.000C>G, p.Arg000Cys (variant of uncertain significance) and c.000C>G, p.Arg000Cys (variant of uncertain significance)
Inheritance	The two *NEB* gene variants have been inherited from both of you. Patient has inherited the c.000C>G, p.Arg000Cys from Mum and the c.000C>G, p.Arg000Cys from Dad.
What happens next	**Clinical recommendations:** *if applicable, can be deleted if not*<br>**Data storage and re-analysis:** Your and Patient's genomic data will be stored securely and can be re-analysed in the future if new clinical questions arise.
Your genetic team	We will work together with the other medical teams involved in Patient's care.<br>**Clinical geneticist:**<br>**Genetic counsellor:**<br>**Genetics follow up:**
Community supports	Further resources and community support networks:<br>• Genomics Info - genomicsinfo.org.au<br>• SWAN Australia - swanaus.org.au<br>• Genetic Alliance Australia - geneticalliance.org.au<br>• MedlinePlus Genetics - medlineplus.gov/genetics<br>*Scan the QR code for more information on genomics*

Patient Name's Genomic Test Results

Family Report Issued: dd/mm/yyyy

Parents' names	Parents' names<br>Study ID: A Testing Laboratory:<br>Sample IDs: Patient – 22W , Mum – 22W , Dad – 22W
Reason for test	Unexplained seizures in Patient
About the test	We performed a '**trio whole genome sequencing' (trio WGS)** test. This test examines your and Patient's genetic information to try and find a cause for Patient's condition. You can find links to more information about this test at the bottom of this document.
Patient's result	**No genetic diagnosis was made**
Possible reasons for result	• The cause of Patient's seizures may not be genetic<br>• The cause of Patient's seizures may be genetic, but<br>o the particular gene change causing his/her seizures may be difficult to detect and interpret with current technology and knowledge<br>o may be due to a change in a gene that is yet to be linked to health problems
What happens next	**Clinical recommendations:** *if applicable, can be deleted if not*<br>**Data storage and re-analysis:** Your and Patient's genomic data will be stored securely and can be re-analysed in the future if new clinical questions arise.
Your genetic team	We will work together with the other medical teams involved in Patient's care.<br>**Clinical geneticist:**<br>**Genetic counsellor:**<br>**Genetics follow up:**
Community supports	Further resources and community support networks:<br>• Genomics Info - genomicsinfo.org.au<br>• SWAN Australia - swanaus.org.au<br>• Genetic Alliance Australia - geneticalliance.org.au<br>• MedlinePlus Genetics - medlineplus.gov/genetics<br>*Scan the QR code for more information on genomics*

Supplementary Notes 2. Family survey tool.

Section 1: Demographics

This section asks questions about yourself. This can be used to help understand if certain trends in responses are linked to being in a particular group. This could be categorised by location, age, or what language you speak.

Note: as some demographic data could identify individual respondents in areas with few acute care units, we will combine data for some analyses.

1) What is your gender? *Please select one option.*

- ❑ Male
- ❑ Female
- ❑ Other
- ❑ Prefer not to answer

  a. If other, please specify……

2) What is your age? (years)......

3) What is your home postcode?......

4) What is the highest level of education you have completed? *Please select one.*

- ❑ Year 11 or below / Certificate 3
- ❑ Year 12 or equivalent / Certificate 4
- ❑ Bachelor degree
- ❑ Graduate degree
- ❑ Postgraduate degree
- ❑ Other (please specify)……

5) What is the combined income of all adults (including you) in your household per year before tax? *Please select one option.*

- ❑ Less than $4,900
- ❑ $5,000 - $9,999
- ❑ $10,000 - $19,999
- ❑ $20,000 - $29,999
- ❑ $30,000 - $39,999
- ❑ $40,000 - $49,999
- ❑ $50,000 - $59,999
- ❑ $60,000 - $69,999
- ❑ $70,000 - $79,999
- ❑ $80,000 - $89,999
- ❑ $90,000 - $119,999
- ❑ $120,000 - $149,999
- ❑ $150,000 or more
- ❑ Prefer not to answer

6) What is your current marital status?

- ❑ Married
- ❑ De facto (living with a partner)
- ❑ Divorced/separated
- ❑ Widowed
- ❑ Never married
- ❑ Other (specify)

  a) If other, please specify…

7) How many children do you have?

- ❑ 0
- ❑ 1
- ❑ 2
- ❑ 3
- ❑ 4
- ❑ 5
- ❑ 6
- ❑ >6

  a) How many of your children are 15 years or younger?

8) Do you have private health insurance?

- ❑ Yes
- ❑ No
- ❑ Unsure

9) **Is English the main language you use?** *Please select one option.*

- ❑ Yes
- ❑ No

a. **If no, do you need assistance reading English?**

- ❑ Yes
- ❑ No

10) **Do you plan on having more children?** *Please select one option.*

- ❑ Yes in the next 2 years
- ❑ Yes in more than 5 years
- ❑ I am not currently planning to have more children
- ❑ Yes in the next 5 years
- ❑ Yes but I'm not sure when
- ❑ Unsure

Section 2: Genomic Medicine and your Child's Family Report

We are gathering information about your experiences so far with genetic testing and genomic medicine. We will ask you to reflect and comment on your experiences with genomic testing, the layout of the family report, the information available on the family report, how you best understand information, and any recommendations you have to add to the report.

EXPERIENCES WITH GENOMIC TESTING

11) **Before the ultra-rapid genomic testing for your child, did you have any experience with genetic conditions, e.g. personal history, family history, general knowledge?** *Please select one option.*

- ❑ Yes
- ❑ No
- ❑ Unsure

a. **If yes or unsure, please describe, including the name of the genetic condition if you know it……**

12) **Before the ultra-rapid genomic testing for your child, did you have any experience with genetic testing, whether through a GP, specialist, genetics clinic, or an online test, e.g., non-invasive prenatal screening/testing (NIPS/NIPT), carrier/reproductive screening, ancestry testing, etc.?** *Please select one option.*

- ❑ Yes
- ❑ No
- ❑ Unsure

a. **If yes or unsure, which genetic testing have you had experience with?** *Please select all that apply.*

- ❑ Non-invasive prenatal screening/testing (NIPS/NIPT) → *reveal **question c.***
- ❑ Ancestry testing
- ❑ Pharmacogenomic testing
- ❑ Carrier/reproductive screening → *reveal **question c.***
- ❑ Nutrigenomic testing
- ❑ Other→*reveal **question b. and c.***

b. **If other, please list…**

c. **Have you paid for any of these tests?**

- ❑ Yes
- ❑ No
- ❑ Unsure

13) **Which of the following best describes the outcome of your child's ultra-rapid genomic test?** *Please select one option.*

- ❑ A genetic diagnosis was made
- ❑ More than one genetic diagnosis was made
- ❑ Not sure
- ❑ A partial genetic diagnosis was made (not all of my child's features were explained by the genetic diagnosis)
- ❑ A genetic diagnosis was not made

14) **Do you recall receiving a family report with the outcome of your child's ultra-rapid genomic test?** *Please select one option.*

- ❑ Yes
- ❑ No → *SURVEY STOP "This survey asks for feedback about the family report. Please contact [relevant Genetic Counsellor] for a copy of the report for your child's ultra-rapid genomic test."*
- ❑ Unsure → *SURVEY STOP "This survey asks for feedback about the family report. Please contact [relevant Genetic Counsellor] for a copy of the report for your child's ultra-rapid genomic test."*

LAYOUT OF THE FAMILY REPORT

15) **How easy was it to find the result of your child's ultra-rapid genomic test in the family report?** *Please select one option.*

❑ Not at all easy ❑ Not so easy ❑ Neutral ❑ Easy ❑ Very easy

16) **How helpful was the family report in understanding the result of your child's ultra-rapid genomic test?** *Please select one option.*

❑ Not at all helpful ❑ Not so helpful ❑ OK ❑ Helpful ❑ Very helpful

17) **How satisfied were you with the general format (layout and style) of the family report?** *Please select one option.*

❑ Not at all satisfied ❑ Not so satisfied ❑ Neutral ❑ Satisfied ❑ Very satisfied

18) **How satisfied were you that the family report was structured in a logical manner?** *Please select one option.*

❑ Not at all satisfied ❑ Not so satisfied ❑ Neutral ❑ Satisfied ❑ Very satisfied

19) **How helpful were visual aids (e.g. pictures, bolded text, section headings, etc.) in helping you understand the information in the family report?** *Please select one option.*

❑ Not at all helpful ❑ Not so helpful ❑ OK ❑ Helpful ❑ Very helpful

a. **Please feel free to comment……**

INFORMATION AVAILABLE IN THE FAMILY REPORT

20) How easy is it to understand the language used in the family report? *Please select one option.*

❑ Not at all easy ❑ Not so easy ❑ Neutral ❑ Easy ❑ Very easy

21) Where medical terms are used in the family report, were they explained in a clear manner? *Please select one option.*

❑ Yes ❑ No → *reveal* ***question a.*** ❑ Unsure → *reveal* ***question a.***

a. If no or unsure, please explain which terms weren't explained clearly and elaborate if you wish……

22) Does the family report contain any unnecessary information? *Please select one option.*

❑ Yes → *reveal* ***question a.*** ❑ No ❑ Unsure→ *reveal* ***question a.***

a. If yes or unsure, please give examples of what information was unnecessary……

[Q21 only for VUS or null result reports that include explanation of test limitations; piping from patient database:]

23) Did your family report explain any limitations of the test? *Please select one option.*

❑ Yes ❑ No ❑ Unsure

24) Did you find it helpful to have a list of which genetic health professionals (your genetic team) are involved in your child's care in the family report? *Please select one option.*

❑ Not at all helpful ❑ Not so helpful ❑ OK ❑ Helpful ❑ Very helpful

25) How easy was it to find sources of further information on the family report? *Please select one option.*

❑ Not at all easy ❑ Not so easy ❑ Neutral ❑ Easy ❑ Very easy

a. Please feel free to comment if there are other types of information you would find helpful……

26) Using the information in the family report, if you were to have another child, what is the chance that the condition would occur again? *Please select one option.*

❑ Less than 1 in 100 (less than 1%) ❑ Less than 1 in 10 (up to 10%) ❑ 1 in 4 (25%) ❑ 1 in 2 (50%) ❑ 3 in 4 (75%) ❑ Definite (100%)

[Q25 will not appear for respondents who chose 'I'm not currently planning to have more children' in Q8:]

27) Using the information in the family report, do you feel you have enough information for future family planning? *Please select one option.*

❑ Yes ❑ No ❑ Unsure

a. If no or unsure, please explain……

28) **Using the information on the family report, would you/have you felt confident explaining the result of your child's ultra-rapid genomic test to someone else e.g. family or friends?** *Please select one option.*

❑ Yes ❑ No ❑ Unsure

a. **If no or unsure, please explain……**

29) **Using the information in the family report, do you feel confident explaining to other family members whether or not they have a chance of being affected by the same condition as your child and/or having a child with the same condition?** *Please select one option.*

❑ Yes ❑ No ❑ Unsure

a. **If no or unsure, please explain……**

30) **Who have you have shared this report with?** *Please select all that apply and provide details of their relationship to you e.g. aunt, social worker. Please do* ***not*** *provide individual names or contact details.*

❑ Other Health Professionals ❑ Friends ❑ No one

❑ Family ❑ Other (specify)

a. **If other, please specify……**

HOW YOU BEST UNDERSTAND INFORMATION

31) **When something is explained to you, is it easier to understand fractions (e.g., 'there's a 1 in 2 (1/2) chance that the coin will be heads') or percentages (e.g., 'there's a 50% chance that the coin will be heads')?** *Please select one option.*

❑ Fractions ❑ Percentages ❑ No difference ❑ Not sure

32) **When reading or watching the news, how helpful do you find tables and graphs to explain parts of the story?** *Please select one option.*

❑ Not at all helpful ❑ Not so helpful ❑ OK ❑ Helpful ❑ Very helpful

33) **When you hear a weather forecast, do you prefer predictions using percentages (e.g., 'there will be a 20% chance of rain today') or predictions using only words (e.g., 'there is a small chance of rain today')?** *Please select one option.*

❑ Percentages ❑ Words ❑ No difference ❑ Not sure

34) **Do you have any suggestions or comments about the family report that would help us improve it?** …………

Supplementary Notes 3. Clinician survey tool.

Section 1: About you

1. **Where do you work?** *Please select all hospitals that apply.*
   - ❑ ACT - The Canberra Hospital
   - ❑ NSW - Children's Hospital Westmead
   - ❑ NSW - John Hunter Children's Hospital
   - ❑ NSW - Royal Prince Alfred
   - ❑ NSW - Royal Hospital for Women Randwick
   - ❑ NSW - Sydney Children's Hospital Randwick
   - ❑ NSW - Westmead Hospital (adult)
   - ❑ NT - The Royal Darwin Hospital
   - ❑ QLD - Queensland Children's Hospital
   - ❑ QLD - Royal Brisbane and Women's Hospital
   - ❑ SA - Women's and Children's Hospital
   - ❑ TAS - The Royal Hobart Hospital
   - ❑ VIC - Royal Children's Hospital
   - ❑ VIC - Monash Health
   - ❑ VIC - Royal Women's Hospital
   - ❑ WA - King Edward Memorial Hospital
   - ❑ WA - Perth Children's Hospital
   - ❑ Other
     - a. **If other, please specify:**

2. **What is your primary role?** *Please select one option.*

   ○ Clinical Geneticist ○ Clinical Genetics trainee ○ Genetic Counsellor

3. **How many years of professional experience in clinical genetics do you have?** *Please select one option.*

   ○ <5 ○ 6-10 ○ 11-15 ○ 16-20 ○ 21-25 ○ >25

4. **How many families have you seen as part of the Acute Care Genomics program <u>from 2020 onwards</u>?** *Please select one option.*

   ○ 1-2 ○ 3-5 ○ 6-10 ○ >10

Section 2: Your feedback on the plain language family report

This survey asks for feedback on the Acute Care Genomics plain language family report. See below for an example of the family report:

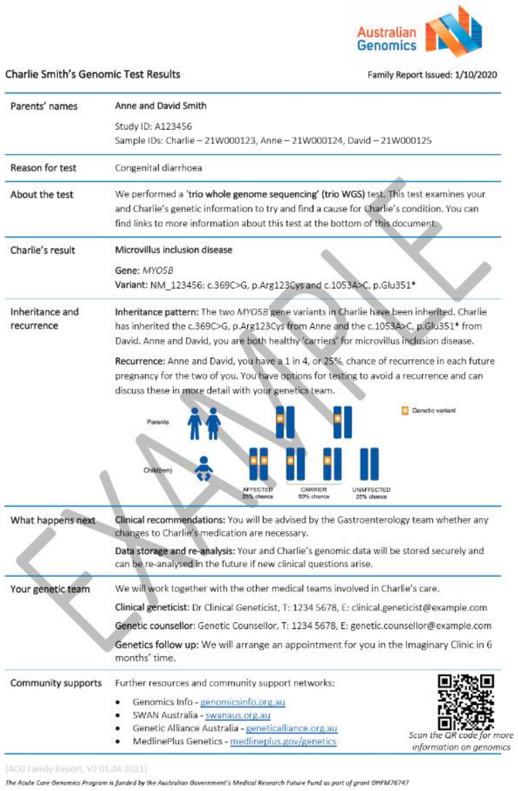

Charlie Smith's Genomic Test Results — Family Report Issued: 1/10/2020

Parents' names	**Anne and David Smith**<br>Study ID: A123456<br>Sample IDs: Charlie – 21W000123, Anne – 21W000124, David – 21W000125
Reason for test	Congenital diarrhoea
About the test	We performed a **'trio whole genome sequencing' (trio WGS)** test. This test examines your and Charlie's genetic information to try and find a cause for Charlie's condition. You can find links to more information about this test at the bottom of this document.
Charlie's result	**Microvillus inclusion disease**<br>**Gene:** *MYO5B*<br>**Variant:** NM_123456: c.369C>G, p.Arg123Cys and c.1053A>C, p.Glu351*
Inheritance and recurrence	**Inheritance pattern:** The two *MYO5B* gene variants in Charlie have been inherited. Charlie has inherited the c.369C>G, p.Arg123Cys from Anne and the c.1053A>C, p.Glu351* from David. Anne and David, you are both healthy 'carriers' for microvillus inclusion disease.<br>**Recurrence:** Anne and David, you have a 1 in 4, or 25%, chance of recurrence in each future pregnancy for the two of you. You have options for testing to avoid a recurrence and can discuss these in more detail with your genetics team.
What happens next	**Clinical recommendations:** You will be advised by the Gastroenterology team whether any changes to Charlie's medication are necessary.<br>**Data storage and re-analysis:** Your and Charlie's genomic data will be stored securely and can be re-analysed in the future if new clinical questions arise.
Your genetic team	We will work together with the other medical teams involved in Charlie's care.<br>**Clinical geneticist:** Dr Clinical Geneticist, T: 1234 5678, E: clinical.geneticist@example.com<br>**Genetic counsellor:** Genetic Counsellor, T: 1234 5678, E: genetic.counsellor@example.com<br>**Genetics follow up:** We will arrange an appointment for you in the Imaginary Clinic in 6 months' time.
Community supports	Further resources and community support networks:<br>• Genomics Info - genomicsinfo.org.au<br>• SWAN Australia - swanaus.org.au<br>• Genetic Alliance Australia - geneticalliance.org.au<br>• MedlinePlus Genetics - medlineplus.gov/genetics<br>*Scan the QR code for more information on genomics*

[ACG Family Report, V2 01.04.2021]

The Acute Care Genomics Program is funded by the Australian Government's Medical Research Future Fund as part of grant GHFM76747

USE OF THE FAMILY REPORT

5. Have you used a family report with any families from the Acute Care Genomics program? *Please select one option.*

○ Yes ○ No → ***EXIT:*** Thank you for your interest in our study but this survey is only for health professionals who have used the family report.

6. How have you used the family report(s)? *Please select all that apply.*

- ❑ At the start of the result disclosure consultation, to guide the discussion
- ❑ During the result disclosure consultation, to help families' understanding and recollection
- ❑ At the end of the result disclosure consultation, to give families a written/visual record to take as a summary
- ❑ Other
  - **a. If other, please tell us more…**

7. How helpful is the family report as part of the result disclosure process? *Please select one option.*

○ Not at all helpful ○ Not so helpful ○ OK ○ Helpful ○ Very helpful

a. Please feel free to comment on the helpfulness of the family report…

8. **Have you distributed a family report to anyone else apart from the family?** E.g., Intensive care team
   - ○ Yes ○ No ○ Unsure
   - a. **If yes, who did you distribute it to and why? …**

9. **Have you used the family report templates outside the Acute Care Genomics program?**
   - ○ Yes ○ No ○ Unsure
   - a. **If yes, please tell us more…**

LAYOUT OF THE FAMILY REPORT

10. **How easy was it to find the result of the ultra-rapid genomic test in the family report?** *Please select one option.*
    - ○ Not at all easy ○ Not so easy ○ Neutral ○ Easy ○ Very easy

11. **How satisfied were you with the general format (layout and style) of the family report?** *Please select one option.*
    - ○ Not at all satisfied ○ Not so satisfied ○ Neutral ○ Satisfied ○ Very satisfied

12. **How satisfied were you that the family report was structured in a logical manner?** *Please select one option.*
    - ○ Not at all satisfied ○ Not so satisfied ○ Neutral ○ Satisfied ○ Very satisfied

13. **How helpful were visual aids (e.g., pictures, bolded text, section headings, etc.) as part of the result disclosure discussion?** *Please select one option.*
    - ○ Not at all helpful ○ Not so helpful ○ OK ○ Helpful ○ Very helpful

14. **Please feel free to comment on any aspect of the layout…**

INFORMATION AVAILABLE IN THE FAMILY REPORT

15. **How easy do you think it is for families to understand the language used in the family report?** *Please select one option.*
    - ○ Not at all easy ○ Not so easy ○ Neutral ○ Easy ○ Very easy

16. **Where medical terms are used in the family report, do you think they are explained in a sufficiently clear manner for families?** *Please select one option.*
    - ○ Yes ○ No ○ Unsure
    - a. **If no or unsure, please explain which terms aren't explained clearly and elaborate if you wish…**

17. **Does the family report contain any unnecessary information for families?** *Please select one option.*
    - ○ Yes ○ No ○ Unsure
    - a. **If yes or unsure, please give examples of what information was unnecessary……**

18. **Did you modify the family report beyond adding the details of the genetics team?** E.g., community supports, clinical recommendations.

- ○ Yes ○ No ○ Unsure

   a. **If yes, how easy was it to modify the family report?**

- ○ Not at all easy ○ Not so easy ○ Neutral ○ Easy ○ Very easy

   b. **What did you modify and why? …**

FINAL COMMENTS

19. **Please feel free to provide any other comments about the family report.** For example, any differences in how you used the report for informative or uninformative results.

Supplementary Table 1. Demographics of family respondents.

**Gender (n=51)**	**n (%)**
female	40 (78)
male	11 (22)
**Age (n=49)**	**mean (range)**
in years	34.9 (22-52)
**Location (state/territory) (n=48)**	**n (%)**
Australian Capital Territory	0 (0)
New South Wales	15 (31)
Northern Territory	0 (0)
Queensland	2 (4)
South Australia	4 (8)
Tasmania	1 (2)
Victoria	24 (50)
Western Australia	2 (4)
**Highest level of education (n=50)**	**n (%)**
secondary	16 (32)
post-secondary	34 (68)
**Income (centiles) (n=49)**	**n (%)**
0-20% (< AU$38,896)	5 (10)
20-40% (AU$38,897 to AU$69,524)	6 (12)
40-60% (AU$69,525 to AU$109,304)	11 (22)
60-80% (AU$109,305 to AU $168,688)	6 (12)
80-100% (>AU$168,689)	19 (39)
prefer not to say / missing	2 (4)
**English as main language (n=51)**	**n (%)**
yes	44 (86)
no	7 (17)
**If English not main language – any assistance needed reading English (n=7)**	**n (%)**
yes	0 (0)
no	7 (100)
**Planning more children (n=51)**	**n (%)**
Yes in the next 2 years	8 (16)
Yes in the next 5 years	3 (6)
Yes in more than 5 years	0 (0)
Yes but I'm not sure when	9 (8)
I am not currently planning to have more children	0 (0)
Unsure	9 (18)

Supplementary Table 2. Demographics of clinician respondents (n=57).

**Location (state/territory)**	**n (%)**
Australian Capital Territory	0 (0)
New South Wales	18 (32)
Northern Territory	0 (0)
Queensland	7 (12)
South Australia	3 (5)
Tasmania	2 (4)
Victoria	23 (40)
Western Australia	4 (7)
**Years of professional experience in clinical genetics**	**n (%)**
<5	22 (39)
6-10	10 (18)
11-15	15 (26)
16-20	5 (9)
21-25	3 (5)
>25	2 (4)
**Number of families seen in ACG study**	**n (%)**
1-2	9 (16)
3-5	15 (26)
6-10	21 (37)
> 10	11 (19)
prefer not to say / missing	1 (2)

Supplementary Table 3. Means for family and clinician responses to five-point Likert scale questions regarding layout, content, and use of ‘family reports’.

	respondents	n	mean	SD
**Layout of ‘family reports’**				
How easy was it to find the result of your child's ultra-rapid genomic test in the family report?	family	40	4.33	0.62
How satisfied were you with the general format (layout and style) of the family report?	family	40	4.18	0.64
How satisfied were you that the family report was structured in a logical manner?	family	39	4.26	0.59
How helpful were visual aids (e.g., pictures, bolded text, section headings, etc.) in helping you understand the information in the family report?	family	40	4.18	0.68
How easy was it to find sources of further information on the family report?	family	37	3.81	0.74
How easy was it to find the result of the ultra-rapid genomic test in the family report?	clinician	53	4.57	0.50
How satisfied were you with the general format (layout and style) of the family report?	clinician	53	4.60	0.49
How satisfied were you that the family report was structured in a logical manner?	clinician	52	4.60	0.50
How helpful were visual aids (e.g., pictures, bolded text, section headings, etc.) as part of the result disclosure discussion?	clinician	53	4.40	0.82
**Content of ‘family reports’**				
How helpful was the family report in understanding the result of your child's ultra-rapid genomic test?	family	39	3.97	0.74
How easy is it to understand the language used in the family report?	family	40	4.05	0.81
How helpful was it to have a list of which genetic health professionals (your genetic team) are involved in your child's care in the family report?	family	39	4.18	0.76
How easy do you think it is for families to understand the language used in the family report?	clinician	52	4.17	0.55
**Use of ‘family reports’**				
How easy was it to modify the family report?	clinician	24	4.25	0.53
How helpful is the family report as part of the result disclosure process?	clinician	53	4.49	0.61

Supplementary Table 4. Final survey questions mapped to constructs: personal characteristics of respondent, 'family report' layout, content, and use. Survey question sources were: [a] Brett *et al.*, 2020 (questions used directly or modified); [b] study investigators (questions crafted by study investigator team); [c] Recchia *et al.*, 2020 (questions used directly or modified); [d] Nisselle *et al.*, 2019 (questions used directly or modified).

Family survey	Clinician survey	Survey question	Source	Personal charact -eristics	Report layout	Report content	Report use
X		What gender are you?	a	X			
X		What is your age? (years)	a	X			
X		What is your home postcode?	a	X			
X		What is the highest level of education you have completed?	a	X			
X		What is the combined income of all adults (including you) in your household per year before tax?	a	X			
X		What is your current marital status?	a	X			
X		How many children do you have?	a	X			
X		Do you have private health insurance?	b	X			
X		Is English the main language you use?	b	X			
X		Do you plan on having more children?	b	X			
X		Before the ultra-rapid genomic testing for your child, did you have any experience with genetic conditions, e.g. personal history, family history, general knowledge?	b	X			
X		Before the ultra-rapid genomic testing for your child, did you have any experience with genetic testing, whether through a GP, specialist, genetics clinic, or an online test, e.g., non-invasive prenatal screening/testing (NIPS/NIPT), carrier/reproductive screening, ancestry testing, etc.?	b	X			
X		Which of the following best describes the outcome of your child's ultra-rapid genomic test?	c	X			
	X	Where do you work?	d	X			
	X	What is your primary role?	d	X			
	X	How many years of professional experience in clinical genetics do you have?	d	X			

Family survey	Clinician survey	Survey question	Source	Personal charact-eristics	Report layout	Report content	Report use
	X	How many families have you seen as part of the Acute Care Genomics program from 2020 onwards?	b	X			
X		Do you recall receiving a family report with the outcome of your child's ultra-rapid genomic test?	b	X			
	X	Have you used a family report with any families from the Acute Care Genomics program?	b				X
	X	How have you used the family report(s)?	b				X
	X	How helpful is the family report as part of the result disclosure process?	b				X
	X	Have you distributed a family report to anyone else apart from the family?	b				X
	X	Have you used the family report templates outside the Acute Care Genomics program?	b				X
X	X	How easy was it to find the result of [your child's/the] ultra-rapid genomic test in the family report?	c		X		
X		How helpful was the family report in understanding the result of your child's ultra-rapid genomic test?	c			X	
X	X	How satisfied were you with the general format (layout and style) of the family report?	c		X		
X	X	How satisfied were you that the family report was structured in a logical manner?	c		X		
X	X	How helpful were visual aids (e.g. pictures, bolded text, section headings, etc.) in helping you understand the information in the family report?	b		X		
X	X	How easy [is it/do you think it is for families] to understand the language used in the family report?	c			X	
X	X	Where medical terms are used in the family report, [were they/do you think they are] explained in a clear manner [for families]?	b			X	

Family survey	Clinician survey	Survey question	Source	Personal charact -eristics	Report layout	Report content	Report use
X	X	Does the family report contain any unnecessary information?	b			X	
X		Did your family report explain any limitations of the test?	b			X	
X		Did you find it helpful to have a list of which genetic health professionals (your genetic team) are involved in your child's care in the family report?	b			X	
X		How easy was it to find sources of further information on the family report?	b		X		
X		Using the information in the family report, if you were to have another child how likely is it that the condition would occur again?	c			X	X
X		Using the information in the family report, do you feel you have enough information for future family planning?	c			X	X
X		Using the information in the family report, would you/do you feel confident explaining the result of your child's ultra-rapid genomic test to someone else e.g. family or friends?	c			X	X
X		Using the information in the family report, do you feel confident explaining to other family members whether or not they have a chance of being affected by the same condition as your child and/or having a child with the same condition?	c			X	X
X		Who have you have shared this report with?	b				X
	X	Did you modify the family report beyond adding the details of the genetics team?	b				X
X		When something is explained to you, is it easier to understand fractions (e.g., 'there's a 1 in 2 (1/2) chance that the coin will be heads') or percentages (e.g., 'there's a 50% chance that the coin will be heads')?	c	X			
X		When reading or watching the news, how helpful do you find tables and graphs to explain parts of the story?	c	X			

Family survey	Clinician survey	Survey question	Source	Personal charact -eristics	Report layout	Report content	Report use
X		When you hear a weather forecast, do you prefer predictions using percentages (e.g., ‘there will be a 20% chance of rain today’) or predictions using only words (e.g., ‘there is a small chance of rain today’)?	c	X			
X		Do you have any suggestions or comments about the family report that would help us improve it?	c		X	X	X
	X	Please feel free to provide any other comments about the family report.	c		X	X	X
