# Supplementary material for: Co-design, implementation, and evaluation of plain language genomic test reports
Source: NPJ Genom Med. 2022 Oct 22;7:61. doi: 10.1038/s41525-022-00332-x (PMC9588009; doi:10.1038/s41525-022-00332-x)
Supplement: Supplementary file 2 — Reporting Summary [file 41525_2022_332_MOESM2_ESM.pdf]

nature portfolio

Corresponding author(s): Gemma Brett

Last updated by author(s): Oct 7, 2022

Reporting Summary

Nature Portfolio wishes to improve the reproducibility of the work that we publish. This form provides structure for consistency and transparency in reporting. For further information on Nature Portfolio policies, see our Editorial Policies and the Editorial Policy Checklist.

Statistics

For all statistical analyses, confirm that the following items are present in the figure legend, table legend, main text, or Methods section.

n/a	Confirmed	
☐	☒	The exact sample size (*n*) for each experimental group/condition, given as a discrete number and unit of measurement
☐	☒	A statement on whether measurements were taken from distinct samples or whether the same sample was measured repeatedly
☒	☐	The statistical test(s) used AND whether they are one- or two-sided<br>*Only common tests should be described solely by name; describe more complex techniques in the Methods section.*
☒	☐	A description of all covariates tested
☒	☐	A description of any assumptions or corrections, such as tests of normality and adjustment for multiple comparisons
☐	☒	A full description of the statistical parameters including central tendency (e.g. means) or other basic estimates (e.g. regression coefficient) AND variation (e.g. standard deviation) or associated estimates of uncertainty (e.g. confidence intervals)
☒	☐	For null hypothesis testing, the test statistic (e.g. *F*, *t*, *r*) with confidence intervals, effect sizes, degrees of freedom and *P* value noted<br>*Give P values as exact values whenever suitable.*
☒	☐	For Bayesian analysis, information on the choice of priors and Markov chain Monte Carlo settings
☒	☐	For hierarchical and complex designs, identification of the appropriate level for tests and full reporting of outcomes
☒	☐	Estimates of effect sizes (e.g. Cohen's *d*, Pearson's *r*), indicating how they were calculated

Our web collection on statistics for biologists contains articles on many of the points above.

Software and code

Policy information about availability of computer code

Data collection	Survey responses were collected and managed using REDCap electronic data capture tools hosted at the Murdoch Children's Research Institute, Melbourne, Australia
Data analysis	Data were interrogated via descriptive statistical analyses conducted using Stata 17

For manuscripts utilizing custom algorithms or software that are central to the research but not yet described in published literature, software must be made available to editors and reviewers. We strongly encourage code deposition in a community repository (e.g. GitHub). See the Nature Portfolio guidelines for submitting code & software for further information.

Data

Policy information about availability of data

All manuscripts must include a data availability statement. This statement should provide the following information, where applicable:
- Accession codes, unique identifiers, or web links for publicly available datasets
- A description of any restrictions on data availability
- For clinical datasets or third party data, please ensure that the statement adheres to our policy

Plain language genomic test report templates and survey instruments are available as supplementary information. Detailed data regarding the survey responses are available upon individual request to gemma.brett@vcgs.org.au.

Human research participants

Policy information about studies involving human research participants and Sex and Gender in Research.

Reporting on sex and gender	gender was self reported
Population characteristics	Inclusion criteria for 'family respondents' were families issued a plain English report through the Acute Care Genomics study October 2020 to November 2021. Exclusion criteria were families who withdrew from the study, requested not to receive surveys, did not provide an email address, or if involved genetic health professionals indicated the family was highly distressed. Inclusion criteria for 'clinician respondents' were genetic health professionals caring for patients undergoing urGS via the Acute Care Genomics study.
Recruitment	Family survey invitations were sent 12 weeks after result disclosure, distributed to primary contact parent/guardian email addresses. Clinician survey invitations were sent via email, with three reminders over two months.
Ethics oversight	Melbourne Health; Royal Children's Hospital

Note that full information on the approval of the study protocol must also be provided in the manuscript.

Field-specific reporting

Please select the one below that is the best fit for your research. If you are not sure, read the appropriate sections before making your selection.

☐ Life sciences ☒ Behavioural & social sciences ☐ Ecological, evolutionary & environmental sciences

For a reference copy of the document with all sections, see nature.com/documents/nr-reporting-summary-flat.pdf

Behavioural & social sciences study design

All studies must disclose on these points even when the disclosure is negative.

Study description	Exploratory survey study, including both quantitative and qualitative questions: multichoice, rating scales, open text responses.
Research sample	Families issued a plain language genomic test report, and clinicians who issued those reports, as part of their participation in the Acute Care Genomics study, October 2020 to November 2021.
Sampling strategy	Family survey invitations were sent 12 weeks after result disclosure, distributed to primary contact parent/guardian email addresses. The survey could only be completed once per family. If the survey was not completed, email reminders were sent after two and four weeks. Clinician survey invitations were sent via email, with three reminders over two months.
Data collection	Survey responses were collected and managed using REDCap electronic data capture tools.
Timing	This paper reports data from responses to family invitations sent March 2021 to February 2022, and clinician invitations sent December 2021 to February 2022.
Data exclusions	All submitted survey responses were included in analysis. As some respondents skipped questions sample size is reported for each question.
Non-participation	One third of 154 families responded (n=51; response rate 33%). Of 107 clinicians, 57 responded (response rate 53%).
Randomization	N/A

Reporting for specific materials, systems and methods

We require information from authors about some types of materials, experimental systems and methods used in many studies. Here, indicate whether each material, system or method listed is relevant to your study. If you are not sure if a list item applies to your research, read the appropriate section before selecting a response.

Materials & experimental systems

n/a	Involved in the study
☒	☐ Antibodies
☒	☐ Eukaryotic cell lines
☒	☐ Palaeontology and archaeology
☒	☐ Animals and other organisms
☒	☐ Clinical data
☒	☐ Dual use research of concern

Methods

n/a	Involved in the study
☒	☐ ChIP-seq
☒	☐ Flow cytometry
☒	☐ MRI-based neuroimaging
